# Old and Unemployable? How Age‐Based Stereotypes Affect Willingness to Hire Job Candidates

**DOI:** 10.1111/josi.12158

**Published:** 2016-03-09

**Authors:** Dominic Abrams, Hannah J. Swift, Lisbeth Drury

**Affiliations:** ^1^ University of Kent

## Abstract

Across the world, people are required, or want, to work until an increasingly old age. But how might prospective employers view job applicants who have skills and qualities that they associate with older adults? This article draws on social role theory, age stereotypes and research on hiring biases, and reports three studies using age‐diverse North American participants. These studies reveal that: (1) positive older age stereotype characteristics are viewed less favorably as criteria for job hire, (2) even when the job role is low‐status, a younger stereotype profile tends to be preferred, and (3) an older stereotype profile is only considered hirable when the role is explicitly cast as subordinate to that of a candidate with a younger age profile. Implications for age‐positive selection procedures and ways to reduce the impact of implicit age biases are discussed.

Global population aging means that between 2000 and 2050 the percentage of the world's population aged over 60 years will double from 11% to 22% (WHO, 2014). In many industrialized nations, this may create an unavoidable obligation to work longer (Feyrer, 2007). However, extending working life means older people may face age stereotypes, resulting in discrimination and exclusion from the labor market (McCann & Giles, 2002). Negative stereotypes that surround older people and older workers (Ng & Feldman, 2012) can harm their performance (Lamont, Swift, & Abrams, 2015) and influence employers’ hiring decisions (Gringart, Helmes, & Speelman, 2005). However, research has yet to examine whether people's *assumptions* about a candidate's age may affect hiring decisions even when there is no disclosure of actual age. In this article, drawing on theories of age stereotypes, social roles and hiring bias, we report a series of studies that investigate how age‐stereotypical characteristics are used as criteria for job hire.

Many countries legislate against age discrimination in the workplace, preventing employers from limiting positions to particular ages (unless objectively justified), and entitling applicants to omit their age from their resumés (Age Discrimination and Employment Act, 1967; Equality Act, 2010). However, information included in job applications and resumés (e.g., including dated qualifications) often enable employers to discern an applicant's age. This could consciously or unconsciously lead to discrimination.

The present studies build on previous research on hiring biases but which has typically varied applicants’ gender or their gender‐stereotypic characteristics to see how this affects judges’ preferences or hiring decisions (Eagly & Karau, 2002). For example, in a series of studies expanding on the “Think Manager—Think Male” effect, Ryan, Haslam, Hersby, and Bongiorno (2011) established a series of traits that are judged to characterize either men or women and discovered that people associate managers of successful companies with masculine traits and managers of unsuccessful companies with feminine traits.

To date, it appears that little research has explored how age‐stereotypic characteristics rather than explicit age may affect judgments of hirability. Indeed, the one study that has examined explicit age‐based candidate preference for an age‐neutral job revealed that younger workers were rated slightly higher on their relevant job qualifications (Cleveland & Landy, 1983). The present work therefore sought to establish characteristics stereotypically associated with younger and older workers and then test whether profiles of candidates possessing these traits influence perceivers’ willingness to hire them.

Based on theories of ageism, which demonstrate that people have implicit preferences for young over old (Levy & Banaji, 2002), and on evidence that youth is more often associated with competence and relatively higher status (Abrams, Russell, Vauclair, & Swift, 2011; Cuddy, Norton, & Fiske, 2005; Fiske, Cuddy, Glick, & Xu, 2002), we expect people to be more willing to hire a candidate with a relatively younger stereotypic profile even though there is no explicit information about that candidate's age. However, given the multidimensional nature of old‐age workplace stereotypes (Dordoni & Argentero, 2015; Swift, Abrams, & Marques, 2012), we assume there may be circumstances that might moderate bias based on tenure (Postuma & Campion, 2008) and status of the position (Abrams et al., 2011).

## Moderators of Age Discrimination in the Workplace

### Short‐ and Long‐Term Goals

One reason why employers may avoid hiring older people for a new position is that older people may provide fewer years of return on any training and investment (Finkelstein & Burke, 1998). Hirers may therefore have greater preference for stereotypically younger candidates if the investment is viewed as long‐ rather than short‐term. In contrast, a review of moderators of workplace age discrimination research revealed evidence for the opposing hypothesis—that older workers are a better long‐term investment because they are less likely to quit (Postuma & Campion, 2008). Study 2 in this research tests these and a third (null) prediction that, because age is not explicit, judges cannot make a rational calculation based on age, and therefore implicit preferences for stereotypically younger candidates would arise regardless of the time frame.

### Role Fit

Social role theory holds that discrimination may occur when there is a mismatch between a person's (gender) stereotypical characteristics and the requirements of the position for which they are applying (Eagly, 1987; Eagly & Karau, 2002). Translated to the age context, older workers are more likely to be discriminated against when there is a stereotypical mismatch between the worker's perceived age and characteristics of a particular position or profession (Postuma & Campion, 2008). Based on societal perceptions that older adults hold lower status than younger adults (Abrams et al., 2011), we expect that stereotypically older candidates will be more likely to be hired if the job itself is lower‐status.

We report pilot work and three empirical studies to test these predictions In each study, people judge two candidates whose ages are not specified but who differ in their age‐stereotypic characteristics. Study 1 examines hiring preferences for these candidates. Study 2 explores whether preferences are affected by the time frame for potential benefit for the employer, and Study 3 examines the effect of job status that is focal for the hiring decision.

## Pilot Studies

Pilot work established two skill sets that would be viewed as stereotypically “young” and “old” by U.S. participants by adapting and adding to the attributes identified originally in U.K. research (Ray, Sharp, & Abrams, 2006; Swift et al., 2013). Participants were recruited and paid to complete the questions via Amazon's Mechanical Turk. The questionnaire presented a set of 20 skills and abilities. Participants were asked to choose whether each ability was “more typical of people in their 20s” (scored 1), “more typical of people in their 60s” (scored 3), or “equally typical of both” (scored 2).

The age stereotypicality of each ability was evaluated by 60 participants (ranging from 18 to 72 years, *M* = 35.1, *SD* = 12.93, 57% Male). Attributes were designated as stereotypical if they were distinctively typical of one group (half or more of respondents assigned it to one age group and fewer than a quarter of respondents assigned it to the other or both age groups). Abilities were defined as neutral if at least half the respondents judged that it applied to both age categories, and no more than 30% selected either age group.

A separate sample of 25 participants (ranging from 18 to 66 years, *M* = 32.9, *SD* = 13.1, 56% Male) rated each attribute on a 7‐point scale (1 = *very negative*, 7 = *very positive*). We then compiled two age‐stereotypic profiles that were matched in terms of mean valence and then added a neutral item to each profile (carefulness and communicativeness).

The two profiles are shown in Table 1. The age categorization of the abilities in the two profiles differed significantly, *t* (59) = 16.12, *p* < .001 and both differed significantly from the scale‐neutral point (2). The mean valence of the two profiles did not differ significantly, *t* (24) = 1.61, *p* = .121. We therefore used these profiles, which are equivalent in valence but differ in age stereotypicality, as the stimuli in the studies that followed.

**Table 1:** Age‐Stereotypic Ability Profiles and Hiring Preferences across Studies

	Person A	Person B
	Settling arguments	Learning new skills
	Understanding other's views	Being creative
	Dealing with people politely	Using new computer technology (e.g., Smartphones)
	Solving crosswords	Rapid decision making
	Being an effective complainer	Being open to new ideas/experiences
	Using a library	Communicativeness
	Carefulness	Using social media (e.g., Facebook)
Pilot *M* (*SD*)		
Age categorization	2.42 (0.30)	1.46 (0.31)
Valence	5.39 (0.47)	5.55 (0.50)
Study 1 hiring preference for Person B (%)		80
Study 2 hiring preference		
Long‐term		85
Short‐term		81
Study 3 hiring preference		
Control		73
Supervised		72
Subordinate		50

Note

Age categorization ranges from 1 = *typical of a person in their 20s* to 3 = *typical of a person in their 60s*. Valence ratings can range from 1 = *very negative* to 7 = *very positive*.

## Study 1

Study 1 tested preferences for these two positive profiles when participants considered each as a candidate for a job. We explicitly stated that the candidates had similar qualifications and neither had previous experience of the job. We expected that the “younger” profile (Candidate B) would be more likely to be selected as a potential job hire.

### Method

#### Participants

Participants were 40 MTURK workers (ranging from 21 to 62 years, *M* = 36.9, *SD* = 11.9, 54% Male). No constraints were placed on participants. The data stopping rule was 40 cases because, from lecture demonstrations that had used a similar stimulus set, we anticipated a large effect size.

#### Procedure and measures

Participants were instructed: “In this study we are asking you to imagine that you are an employer who is looking at applications from two different people that are applying for the same job. As the employer, your goal is to hire someone who will maximize the profits of your company. Your task is quite difficult because there are a lot of candidates who have similar qualifications and none have any previous experience in this kind of job. Each candidate also completed a psychometric questionnaire about their interests, skills and abilities, and this has given you a profile of ways in which each candidate is distinctive from the other candidates. Using this information your task is to select the person that you wish to employ to maximize the profits of your company. To keep these names anonymous, we have labelled these candidates with letters A and B rather than providing their actual names.”

#### Hiring decision

Participants then viewed the two profiles simultaneously before responding to the question: “Who would you hire?” They were asked to select a button to show if they would hire Person A, Person B, or were unsure.

#### Age estimates

On the next screen, participants were then asked to estimate the age of each candidate using a slider scale (from 19 to 81).

### Results

#### Hiring decision

Eighty percent (32) of the participants chose to hire Candidate B (the younger profile). Fifteen percent selected Candidate A and 5% (2) were unsure, χ^2^ = 14.40, *p* < .001 (see Table 1).

Point biserial correlation analyses showed that participants’ age and gender were not significantly related to their candidate choice (*ps* > .70).

#### Age estimates

A repeated measure ANOVA showed that Candidate A was judged to be older (*M* = 36.53, *SD* = 9.76) than Candidate B (*M* = 32.10, *SD* = 9.65), *F* (1, 39) = 4.56, *p* = .039, η_p_^2^ = .105. Moreover, the participants who chose Candidate B estimated Candidate B's age to be lower than did participants who did not choose Candidate B (point biserial *r* = ‐.39, *p* = .012). Finally, multiple regression analysis showed that when participants’ own age and gender and their estimates of Candidate A's age were included as covariates, the relationship between estimates of Candidate B's age and hiring choice remained significant (β = ‐.36, *t* = 2.16, *p* = .038).

In summary, only a minority of participants chose to hire the stereotypically older age profile (A). Participants’ assumed Candidate B was younger and the more they did so, the more they preferred to hire Candidate B, consistent with the idea that implicit age stereotypes affected hiring decisions.

## Study 2

Given the goal of “maximizing profits,” a plausible explanation for hiring a stereotypically younger candidate is based on “rational” cost‐benefit calculations. If participants had long‐term profits in mind in Study 1, the “younger” candidate could work for longer before reaching retirement and provide greater total profit for the company. Alternatively, if participants had short‐term profits in mind, their preference for the younger profile may be because they discounted the stereotypically older candidate's potentially greater long‐term value due to their lower turnover intention (Posthuma & Campion, 2008). To test these possibilities, Study 2 examined whether the selection chances of the stereotypically older profile (Candidate A) would depend on whether the employer's goal was short‐, rather than long‐term profits. However, we noted from Study 1 that the age‐stereotype link generated quite a small explicit difference in age estimates for the two candidates. This makes it less likely that it is the specific age of candidates that affects decisions but rather perceptions of relative age and implicit ageism. In that case, the preference for the “younger” profile may persist regardless of time perspective.

### Method

#### Participants and design

Eighty MTURK workers (ranging from 19 to 70 years, *M* = 35.3, *SD* = 11.7, 60% Male) were recruited as participants. Using random assignment to condition (via Qualtrics software), we presented the profiles for Candidates A and B and defined either short‐ or long‐term objectives.

#### Procedure and measures

In the short‐ and long‐term conditions (respective differences shown in parentheses), participants were instructed as Study 1. However, “maximize the profits of your company” was replaced with “be an ideal worker for the [short term/long term] benefit of your company over [the next financial year/ a number of financial years].” Participants then completed the job hire and age estimation measures as in Study 1.

### Results

#### Hiring decision

Eighty‐three percent of participants selected Candidate B (χ^2^ = 37.33, *p* < .001). Moreover, time frame condition made no difference to the selection of candidates, χ^2^ = 0.34. Eighty‐one percent and 85% chose Candidate B in the short‐ and long‐term conditions, respectively (see Table 1).

#### Age estimations

Candidate B was judged to be significantly younger than Candidate A, repeating the finding from Study 1, *F* (1, 79) = 20.58, η_p_^2^ = .207.

In summary, regardless of whether they were considering hiring for a short‐term or long‐term position, participants strongly preferred a stereotypically younger age profile.

## Study 3

We extended our consideration of the stereotypical status differences between older and younger people. Based on Eagly's Role Theory (1987) and the stereotype content model (Fiske et al., 2002), we considered that the warm/less competent older stereotype would be more compatible with a low‐status role. Therefore, in Study 3, we compared whether specifying a position as low‐status would increase the probability that the stereotypically older candidate (A) would be hired.

Given that Studies 1 and 2 revealed a strong preference for hiring Candidate B, and based on role theory, we wondered if low status per se would be sufficient to make Candidate A attractive. Specifically, whereas stereotype‐based models of ageism have identified that being older (in general) is associated with lower societal status (Abrams et al., 2011; Cuddy et al., 2005), a role‐based interpretation might assume that the low status might only affect a hiring decision if there is certainty that the job position would be subordinate to someone who should have higher status, thereby assuring role fit.

To test this idea, we compared the baseline condition of Study 1 (Control condition) against two alternative scenarios involving a low‐status criterion for hiring. We either specified that the task was to hire a person to occupy a supervisee role (Supervisee condition), or we specified that participants should select which of the two candidates should be supervised by (subordinate to) the other (Subordinate condition). The Supervisee and Subordinate conditions both required participants to select a person to be supervised, but the Subordinate condition involved explicit subordination of one candidate to the other, thus ensuring fulfillment of a comparatively lower‐status role. To explore how participants were thinking about the different roles, we also investigated perceptions of the candidates. If hiring decisions are driven by implicit ageism and only one candidate can be hired, participants should still favor the “younger profile,” even as a supervisee. But this “younger” preference should reduce if the selected candidate will be subordinate to the other because of the less close role fit between being stereotypically younger and a relatively lower status position.

### Method

#### Participants and design

One hundred and fifty MTURK participants (ranging from 19 to 67 years, *M* = 35.6, *SD* = 12.4, 55% Female) were randomly assigned to condition (Control, Supervisee, Subordinate).

#### Procedure and measures

The Control condition instructed participants to hire a candidate to maximize profits, exactly as in Study 1. The Supervisee condition and Subordinate condition instructions (distinguished by a slash in parentheses) were as follows: “In this study we are asking people to imagine that they are an employer who is looking at applications from two different people who are applying for [a job/two jobs]. You will hire [one person/both people] so you must decide which one should be hired to be [supervised /supervised by the other]. As the employer, your goal is to choose which one should be [supervised/the subordinate (supervised)]. The other one [will not be hired/will be the supervisor]. Your task is quite difficult because these have similar qualifications and neither has any previous experience in this kind of job. But both people completed a psychometric questionnaire about their interests, skills and abilities, and this has given you a profile of ways in which each candidate is distinctive from the other. Using this information your task is to select which person should hired to be [supervised/subordinate (supervised)]. The other one will [not be hired/ be the supervisor]. To keep these names anonymous, we have labeled candidates with letters (Person A, and Person B) rather than providing actual names. Your task is to decide whether Person A or Person B should be the one who should be [hired to be supervised /subordinate (supervised)]. Click next to view the profile of each candidate.”

Participants then completed the hiring decision and age estimation measures. In order to understand reasons for hiring decisions, we asked participants to judge how important each attribute was for the job, to evaluate the profiles of the two candidates, and to infer demographic characteristics for the two profiles.

#### Job‐related importance of attributes

Participants were asked how important each of the following attributes was for the job (1 = *not at all important*, 7 = *extremely important*): settling arguments, understanding others’ views, dealing with people politely, solving crosswords, being an effective complainer, using a library, carefulness, learning new skills, being creative, using new computer technology (e.g., smartphones), rapid decision making, openness to new ideas and experiences, communicativeness, using social media (e.g., Facebook), and other (free response). Presentation of all but the last item was randomized.

#### Trait inferences

Participants were asked to rate (1 = *very unlikely*, 5 = *very likely*) whether Person A and Person B were gentle, intelligent, warm, moral, exciting, interesting, admirable, perform well at tasks, have a lot of potential, are resourceful, reliable, loyal, open, efficient, motivated, experienced, needy, financially smart, risk takers, and natural leaders. The presentation order of these characteristics was randomized.

#### Demographic inferences

Participants were asked to indicate whether they thought Person A and/or Person B were male/female, White/Black/Hispanic/Asian, heterosexual/gay or bisexual, religious/nonreligious, American. Order of presentation was randomized.

### Results

#### Hiring decision

Overall, 64.8% selected Candidate B (χ^2^ = 12.75, *p* < .001). However, this proportion varied as a function of condition, χ^2^ (2 df) = 7.38, *p* = .029). Specifically, whereas 73.3% chose Candidate B in the control condition, and 72% in the supervisee condition, this reduced to 50% in the subordinate condition (see Table 1).

#### Age estimates

Candidate B (*M* = 32.42, *SD* = 8.66) was judged as significantly younger than Candidate A (*M* = 37.92, *SD* = 9.66), repeating the findings from Studies 1 and 2, *F* (1,138) = 23.26, *p* < .001, η_p_^2^ = .144. Moreover, estimates of candidates’ ages did not vary by condition, suggesting that differences in hiring decisions were not because the subordinate condition had altered the perceived age difference between the candidates.

Inspection of correlations within conditions indicated that participants in the Control condition who selected Person B were significantly more likely to estimate Person B's age as younger (*r* = ‐.40, *p* = .009). In contrast, participants in the Subordinate condition who selected Candidate B were significantly more likely to estimate Candidate B's age as being older (*r* = .38, *p* = .007). In the Supervisee condition, there was no significant correlation (*r* = .04, *p* = .790). This suggested an interactive effect of condition and perceived age on hiring decisions. To test this possibility, we dummy coded conditions and created interaction terms between the Control condition and estimates of Candidate B's age, and between the Subordinate condition and Candidate B's estimated age. We then conducted a regression analysis to test the effects of participants’ age and gender, Control condition and Subordinate condition, age estimate of Candidate B, and the two interaction terms on whether participants selected Candidate B.

The analysis confirmed that there were no significant effects of participants’ age or gender (βs = ‐.09, ‐.15) or their estimates of Candidate A's age (β = ‐.05). Both the Control condition and the Subordinate condition differed from the means of the alternative conditions (βs = .65, ‐1.04, *p*s = .01, < .001, respectively). More interesting were the Control x estimated age of Candidate B interaction, β = ‐.65, *t* = ‐2.62, *p* = .01, and the Subordinate x age of Candidate B interaction, β = .86, *t* = 3.21, *p* = .002. The addition of these interaction terms increased the *R* ^2^ from .08 to .18, and *F* for the final equation was *F* (7,132) = 4.24, *p* < .001. To summarize this finding, when participants simply had the goal of selecting the best candidate, the younger they estimated Candidate B's age, the more likely they were to select Candidate B. When participants had the goal of selecting which candidate should be subordinate, the older they perceived Candidate B to be the more likely they were to select Candidate B.

We repeated these analyses but with the estimated age of Candidate A as the independent variable, whether Candidate A was chosen as the dependent variable, and estimated age of Candidate B as a covariate. This revealed no effects except a significant Subordinate condition versus other conditions effect (β = .23, *t* = 2.44, *p* = .028), all other *p*s > .10. This simply reflects that finding that Candidate A was more likely to be selected in the Subordinate condition than in other conditions.

#### Job‐related importance of attributes

The job characteristics were averaged into two scores, one for the importance of the characteristics presented in the profile of Candidate A (the older profile) and one for Candidate B (the younger profile.). We conducted a repeated measure ANCOVA (Condition x Profile), with Condition as a between participants factor and Profile (older, younger) as a within participants factor. Participant age and gender were covariates. This revealed no significant effects of the covariates, but a significant effect of Condition, *F* (2,135) = 4.50, *p* = .013, η_p_^2^ = .062, a significant effect of Profile, *F* (1,135) = 8.18, *p* = .005, η_p_^2^ = .057, and a significant Condition x Profile interaction, *F* (2,135) = 5.94, *p* = .003, η_p_^2^ = .081.

Attributes were regarded as less important when no role was specified (*M* = 4.88, *SD* = 0.72), than when the role was either supervised (*M* = 5.18, *SD* = 0.61) or subordinate (*M* = 5.26, *SD* = 0.53). The older profile attributes were regarded as less important (*M* = 4.58, *SD* = 0.87), than the younger profile attributes (*M* = 5.64, *SD* = 0.76). Simple effects tests showed that whereas the importance of the young profile attributes did not differ between conditions, *F* (2,135) = 0.79, *p* = .457, η_p_^2^ = .012, the importance of the older profile attributes did differ, *F* (2,135) = 8.87, *p* < .001, η_p_^2^ = .116. Pairwise comparisons showed that the attributes were accorded less importance in the Control condition (*M* = 4.18, *SD* = 1.05) than in either the Supervisee (*M* = 4.64, *SD* = 0.81) or Subordinate (*M* = 4.92, *SD* = 0.58) conditions (*p*s = .017, < .001, respectively) and that the importance was greater in the Subordinate than in the Supervisee condition (*p* = .062).

#### Trait inferences

The items were averaged into mean positivity ratings for each candidate (alphas > .7) and these were subjected to analysis by ANCOVA. This revealed a significant main effect of Condition, *F* (1,134) = 10.59, *p* < .001, η_p_^2^ = .137, and a marginal interaction, *F* (2,134) = 2.58, *p* = .08, η_p_^2^ = .037). However, the simple effect of Condition was significant only for Candidate A, *F* (1,134) = 10.89, *p* < .001, η_p_^2^ = .140. Candidate A was rated less positively in the Control condition (*M* = 3.25, *SD* = 0.67) than in either the Supervisee condition (*M* = 3.52, *SD* = 0.51, *p* = .02) or the Subordinate condition (*M* = 3.78, *SD* = 0.42, *p* < .001), and less favorably in the Supervisee condition than the Subordinate condition (*p* = .017). In contrast, the simple effect of Condition was nonsignificant for Candidate B, *F* (1,134) = 1.58, *p* = .209, η_p_^2^ = .023, as this candidate was rated equally positively in all conditions (*Ms* = 3.71, 3.82, 3.89, *SDs* = 0.53, 0.50, 0.45, respectively, all pairwise *ps* > .07). Moreover, whereas ratings of A and B differed significantly in both the Control, *F* (1,134) = 16.02, *p* < .001, η_p_^2^ = .107, and the Supervisee condition, *F* (1,134) = 8.87, *p* = .003, η_p_^2^ = .062, they did not differ significantly in the Subordinate condition, *F* (1,134) = 1.33, *p* = .250, η_p_^2^ = .01.

#### Demographic inferences

These data were coded first according to whether or not the candidate was judged to have a majority group characteristic (White, male, American, religious, heterosexual). Repeated measure MANCOVA revealed no significant differences due to Condition, Candidate, or participant gender or age. These scores were factor analyzed for each candidate. Because they all loaded significantly on the first principle component, an average “majority” score was created for each candidate. This score could range from 0 (no majority characteristics) to 1 (entirely majority characteristics). Overall, participants judged that at least half of the candidates’ characteristics were majority memberships (*M* = 0.58, *SD* = 0.28). A repeated measure ANCOVA on this score confirmed the MANCOVA findings and revealed no significant differences due to Condition, candidate, or participant gender or age. These analyses confirm that the profiles differed only in terms of their stereotypical age and were not associated with other major demographic characteristic.

#### Mediation analyses

Because hiring choices differed between the Subordinate and other conditions, we sought to explain why preferences shifted in the Subordinate condition. To simplify analyses, we constructed a difference score for the relative importance of the profile characteristics for the job (Candidate B minus Candidate A), and a difference score for the relative positivity ratings of Candidate B minus the positivity ratings of Candidate A. ANCOVAs showed that these two scores differed significantly between the Subordinate and other conditions.

The relative importance and relative favorability measures were significantly correlated with one another (*r* = .49, *p* < .001), and each was significantly correlated with hiring choice (point biserial *r* = .48, .49, respectively). Given that both could potentially mediate between conditions and hiring decisions, we conducted a parallel mediation analysis using Hayes’ (2013) Process SPSS Macro (model 4 with 5,000 bootstraps), including participant age and gender as covariates.

**Figure 1:**
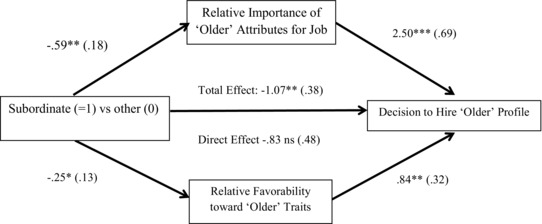
Effect of condition on hiring decisions, mediated by attribute importance and favorability ratings. Note. Indirect effects via relative importance, *B* = ‐.501, *SE* = .25, 95% CI [‐1.11, ‐0.14] and relative favorability, *B* = ‐.633, *SE* = .37, 95% CI [‐1.46, ‐0.07], do not differ from one another, *B* = .132, SE = .43, 95% CI [‐1.12, 0.56].

The covariates were nonsignificant and the total effect of Subordinate condition was significant, *B* = ‐1.07, *SE* = .38, 95% CI [‐1.81, ‐0.33]. There were significant indirect effects of both job importance *B* = ‐.50, *SE* = .25, 95% CI [‐1.11, ‐0.14], and profile ratings, *B* = ‐.63, *SE* = .70, 95% CI [‐1.46, ‐0.07], and the direct effect of Subordinate condition was not significant, *B* = ‐.83, *SE* = .48, 95% CI [‐1.77, 0.11] (see Figure 1).

In summary, the subordinate condition increased participants’ relative favorability toward Candidate A's characteristics, and also their judgments of whether those characteristics were relatively important for the job. These two effects accounted for increased selection of Candidate A.

## Discussion

This research is the first, to our knowledge, to have systematically tested whether exhibiting age‐stereotypic characteristics *per se* may affect a candidate's chances of being hired. We established profiles of age‐stereotyped job characteristics that are more stereotypical of a person in their 20s or in their 60s, respectively. We established that the characteristics, judged without reference to the job context, are judged equally positively. In the studies that followed, we ascribed the profiles to Candidate A (older profile) and Candidate B (younger profile), respectively. Across three studies, these profiles led participants to assume Candidate B was younger than Candidate A. In Study 1, 80% of participants selected the younger profile (B) to maximize their company's profits. Study 2 established that this preference could not be attributed to judges’ adoption of a long‐ or short‐term time frame. Therefore, decisions were unlikely to be rationally based on candidates’ likely cumulative contribution or turnover intention. Candidate B was strongly preferred, regardless of whether the goal was to maximize short‐ or long‐term profits.

Study 3 tested whether role fit accounted for whether the older age profile would be “hirable.” Even when the role involved being supervised, selection of Candidate A only increased when the role was explicitly subordinate to that of the younger profile (B).

Study 3 also examined the perceptions and inferences that participants made about the attributes of the two candidates and what would be necessary for the job. Overall, participants rated Candidate B's characteristics as more important for the job and rated them more positively. Note that the latter finding appears to contradict the idea that the two profiles shared a similar valence. However, whereas the pilot research showed that the characteristics themselves had similar valence, these evaluations were clearly altered when participants considered them as being relevant to a job rather than in a context‐free manner. Importantly, we found that the differences in the job relevance and ratings of the two sets of attributes were significantly lower when participants were considering them for the subordinate role. Moreover, this reduced differentiation also explained, statistically, why participants were willing to select Candidate A, the older profile, when considering a subordinate role.

Taken together, these findings are in line with a social role account (Eagly, 1987) and strongly indicate that job applicants may well be vulnerable to implicit age stereotyping and ageist assumptions that older workers belong in low‐status roles. Ironically, even when an applicant highlights positively valued traits and skills, if mentioning these skills invokes old‐age stereotypes, they could well create implicit beliefs that the candidate is “older” than others, and this could place them at a disadvantage relative to applicants who only highlight their “young” stereotypical attributes.

## Limitations, Future Directions, and Implications for Policy and Practice

These studies are novel and we acknowledge several limitations. International generalizability of the findings has yet to be established because, whilst drawing initially from U.K. evidence, the studies all involve only North American participants. Nor is it known whether the requirement to “maximize profits” affected the level of bias. Many organizations define profit as their primary objective but the salience of other goals (e.g., providing excellent services) might tilt biases in other directions (cf. Finkelstein & Burke, 1998). The decisions of actual managers and recruitment staff may differ if they are motivated to avoid stereotype‐based bias (Singer & Sewell, 1989). More generally, making judges feel more accountable for their decisions (Gordon, Rozelle, & Baxter, 1988), reducing their cognitive busyness (Perry, Kulik, & Bourhis, 1996), or reducing intergenerational resource scarcity (North & Fiske, 2016) may moderate their reliance on stereotypes for hiring decisions.

Based on role theory (Eagly, 1987), we argued that older = lower status. Even if this is not always true for high levels of certain occupations (e.g., judges, surgeons, politicians, CEOs), these older high‐status roles may still involve significant “younger” stereotypic attributes. Thus, even for these roles there may be an advantage in highlighting a higher proportion of such attributes at the selection stages. These are all questions for future research.

Implicit age bias in hiring has policy relevance for individuals, organizations, and society. For individuals, exclusion from the labor market can increase the likelihood of depression and mental health problems for older adults (Aquino, Russell, Cutrona, & Altmaier, 1996; Gallo et al., 2006) whereas there are significant psychological benefits for older people that remain in the workforce (Schooler, Mulatu, & Oates, 1999). If job candidates who present or reveal older‐stereotypic abilities and skills activate recruiters' implicitly ageist hiring preferences, this suggests that both applicants and recruiters should be made aware of these potential biases in order to avoid or challenge them directly. Strategically, candidates could tailor their resumés to display only competence and other stereotypically “young” traits that organizations have preferences for, such as learning new skills, creativity, and competence using technology to mitigate the application of old stereotypes. Although this may actually increase resentment toward older workers in conditions where resources are scarce and if older workers are perceived to violate prescriptive norms (North & Fiske, 2016). Ideally, however, employers would learn to recognize the actual advantages and strengths of both older and younger stereotypical qualities rather than to assume one set is inevitably better.

Even for objectively or stereotypically younger people these findings are troubling. Younger people eventually become older, so the perpetual application of ageist hiring assumptions means that people may approach aging with growing anxiety and dread of age discrimination. This not only poses a stereotype threat that could well harm their actual capacity to perform well at work (Lamont et al., 2015), but has potential to decrease job satisfaction and job commitment (Macdonald & Levy, 2016).

The implication of these findings is that implicit age bias could lead organizations to fail to select the best candidates because of unacknowledged assumptions about candidates’ age. Thus, organizations may well underperform because age inferences are drawn that downplay the strengths of candidates who show relatively more “older” characteristics.

At a societal level, the need to retain people in the workforce longer and to sustain incomes and resources into later old age all mean that biases against “older” abilities and skills will lead to reduced opportunities, greater impoverishment, and ultimately more dependency among the oldest members of society. This research shows that even positive age stereotypes may be a substantial driver of age discrimination in employment.
